# The role of vascular endothelial growth factor and matrix metalloproteinases in canine lymphoma: in vivo and in vitro study

**DOI:** 10.1186/1746-6148-9-94

**Published:** 2013-05-03

**Authors:** Arianna Aricò, Mery Giantin, Maria Elena Gelain, Fulvio Riondato, Stefano Comazzi, Barbara C Rütgen, Sabine E Essler, Mauro Dacasto, Massimo Castagnaro, Luca Aresu

**Affiliations:** 1 Department of Comparative Biomedicine and Food Science, University of Padova, Viale dell'Università 16, Agripolis Legnaro, PD, 35020, Italy; 2 Department of Animal Pathology, Faculty of Veterinary Medicine, University of Torino, Via Leonardo da Vinci 44, Grugliasco, TO, 10095, Italy; 3 Department of Animal Pathology, Public Health and Veterinary Hygiene, Faculty of Veterinary Medicine, University of Milano, Via Celoria 10, Milan, 20133, Italy; 4 Clinical Pathology, Department of Pathobiology, University of Veterinary Medicine Vienna, Veterinärplatz 1, Vienna, 1210, Austria; 5 Institute of Immunology, Department of Pathobiology, University of Veterinary Medicine Vienna, Veterinärplatz 1, Vienna, 1210, Austria

**Keywords:** Dog, Phenotype, Lymphoma, MMPs, TIMPs, VEGF

## Abstract

**Background:**

Canine lymphoma represents the most frequent haematopoietic cancer and it shares some similarities with human non-Hodgkin lymphoma. Matrix metalloproteinases (MMPs) and vascular endothelial growth factor (VEGF) play a coordinated role during invasion and proliferation of malignant cells; however, little is known about their role in canine haematologic malignancies. The aim of this study was to investigate the mRNA and protein expression of VEGF and the most relevant MMPs in canine lymphoma. Lymph node aspirates from 26 B-cell and 21 T-cell lymphomas were collected. The protein expression levels of MMP-9, MMP-2 and VEGF-A were evaluated by immunocytochemistry, and the mRNA levels of MMP-2, MMP-9, MT1-MMP, TIMP-1, TIMP-2, RECK, VEGF-A and VEGF-164 were measured using quantitative RT-PCR.

**Results:**

MT1-MMP, TIMP-1 and RECK mRNA levels were significantly higher in T-cell lymphomas than in B-cell lymphomas. Higher mRNA and protein levels of MMP-9 and VEGF-A were observed in T-cell lymphomas than in B-cell lymphomas and healthy control lymph nodes. A positive correlation was found between MMP-9 and VEGF-A in T-cell lymphomas. Moreover, MMP-9, MT1-MMP, TIMP-1 and VEGF-A were expressed at the highest levels in high-grade T-cell lymphomas.

**Conclusions:**

This study provides new information on the expression of different MMPs and VEGF in canine lymphoma, suggesting a possible correlation between different MMPs and VEGF, immunophenotype and prognosis.

## Background

Canine lymphoma is a heterogeneous group of neoplastic disorders and different subtypes are characterized by different morphology, pathophysiology and clinical behaviour [1,2]. Different reports in human medicine have shown that lymphoproliferative tumours are able to produce extra cellular matrix (ECM)-degrading enzymes with proteolytic activity. In this context, matrix metalloproteinases (MMPs) have been demonstrated to have a role in the pathogenesis of lymphoma and be of prognostic significance [3,4]. MMPs are zinc-dependent enzymes responsible for the degradation of the ECM components, including the basement membrane, a natural barrier with a key role in preventing invasion and migration of tumour cells [5]. Tumour cells can self-produce and secrete MMPs to promote invasion and increase their metastatic potential [6]. Once MMPs are released, their proteolytic activity is controlled by the activation of latent proenzymes and by the inhibition of activated proteinases. These processes are controlled by tissue inhibitor metalloproteinases (TIMPs) that are co-secreted by MMP-producing cells [5]. Among MMPs, MMP-2 and MMP-9 play a critical role in the modification of ECM and tumour invasion. Activation of MMP-2 occurs at the cell surface and is mediated by membrane type-1 MMP (MT1-MMP) [7]. A new MMP inhibitor, reversion-inducing cysteine-rich-protein with Kazal motifs (RECK), was reported to down-regulate different MMPs in dogs [8]. MMP-9 is a functional component of the angiogenic switch during carcinogenesis, increasing the availability of vascular endothelial growth factor (VEGF) in a sort of vicious circle [9]. Recently, VEGF has received considerable attention in canine solid neoplasia [10,11]. We applied the concept of tumour microenvironment to canine leukaemia and demonstrated that VEGF could stimulate new blood vessels growth and increase endothelium permeability for systemic dissemination of leukaemic cells [12]. In human lymphoma, VEGF has two potential roles: increase of angiogenesis and proliferation and/or survival of lymphoma cell induced by autocrine vascular endothelial growth factor receptor-mediated signalling [9].

In a previous work in dogs, we demonstrated that plasma levels of MMP-9 and VEGF are higher in B-cell and T-cell lymphomas with respect to healthy dogs and that their levels significantly decreased in B-cell lymphomas during chemotherapy [13]. The role of these proteins in canine lymphoma is still unclear and only scarce data are available in veterinary medicine [13-16]. The aim of the present study was to assess the gene expression profiles of MMP-2, MMP-9, MT1-MMP, TIMP-1, TIMP-2, RECK, VEGF-A and VEGF-164 and the protein levels of MMP-2, MMP-9 and VEGF-A in canine B and T-cell lymphomas.

## Methods

### Chemicals

The chemicals, reagents and kits used in this study were obtained from the following companies: foetal bovine serum, sodium azide, ammonium chloride, potassium bicarbonate, ethylenediaminetetraacetic acid, methanol and acetone were obtained from Sigma Aldrich (Munich, Germany); RPMI 1640 medium was obtained from Sigma Aldrich (Munich, Germany) or PAA, (Pasching, Austria), fetal calf serum (FCS), penicillin, streptomycin were obtained from PAA, (Pasching, Austria); May–Grunwald–Giemsa was obtained from Merck KGaA (Frankfurt, Germany); L-glutamine was obtained from Mediatech Inc. (Herndon, VA, USA); RNAlater® solution and High Capacity cDNA Reverse Transcription Kit were obtained from Applied Biosystems (Foster City, CA, USA); the RNeasy Mini Kit, RNase-Free DNase set and the QuantiTect Reverse Transcription Kit were obtained from Qiagen (Milan, Italy); the LightCycler 480® Probe Master and the human Universal Probe Library (UPL) probe were obtained from Roche Diagnostics (Basel, Switzerland); primer pairs were obtained from Eurofins MWG Operon (Ebersberg, Germany); and biotinylated anti-mouse secondary antibody, diaminobenzidine substrate, and haematoxylin were obtained from Ventana Medical Systems (Tucson, AZ, USA).

### Caseload, cell lines and classification

Fine-needle aspirates (FNAs) of enlarged lymph nodes obtained from dogs with lymphoma were collected. Samples were sent by the referring veterinarians to the Department of Veterinary Pathology, Hygiene and Health at the University of Milan, and to the Department of Animal Pathology at the University of Turin for diagnostic purposes. The study was approved and granted by MIUR PRIN 2008, protocol 20085MSFH2.

The different subtypes were described according to the updated Kiel classification [17] by considering pleomorphism; cell size; nuclear shape; chromatin density; mitotic index; the number, size and distribution of nucleoli; and the extension and basophilia of the cytoplasm. The immunophenotype was determined by means of flow cytometry with the use of the following monoclonal antibodies: CD45-PEb (clone YKIX716.13, Serotec, Oxford, UK, leukocytes), CD3-FITC (clone CA17.2A12, Serotec, T cells), CD4-FITC (clone YKIX302.9, Serotec, T-helper cells and neutrophils), CD8-PE (clone YCATE55.9, Serotec, T-cytotoxic/suppressors), CD5 (clone YKIX322.3, T-cells), CD21-PE (clone CA21D6 Serotec, mature B cells), CD34-PE (clone 1H6, Pharmingen, Becton Dickinson, San Jose, CA, precursor cells), and CD79a (clone HM57, Dako, Atlanta, GA, all stages of B-cells). Data acquisition was performed by using a FACSCalibur (Becton Dickinson), and the analysis was conducted with a commercially available software (Cell Quest, Becton Dickinson). The expression of specific lineage markers defined the lineages of lymphoma: CD3, CD5, CD4, and/or CD8 for T-cell lymphomas, and CD21 and CD79a for B-cell lymphomas. A positive staining referred to the antigen expression in at least 20% of the gated cells. Samples being characterized by an ambiguous diagnosis, low cellularity or viability were excluded from the present study. Five healthy dogs with no relevant peripheral lymph node alterations served as controls. Informed consent was obtained from all owners according to the regulations of each institutional animal care committee.

Two canine lymphoma cell lines (CLBL-1, B-cell lymphoma [18], and OSW, T-cell lymphoma [19]) were used for the study [18,19]. The cell lines were maintained in RPMI 1640 cell culture medium supplemented with 10% heat inactivated FCS, penicillin 100 U/ml/streptomycin 0.1 mg/ml at 37°C in a humidified atmosphere of 5% CO_2_.

### Sampling procedure

Two samples were obtained from each lymph node FNA for routine cytological assessment and flow cytometry, and for immunocytochemical analysis and total RNA extraction. For flow cytometry, cells were suspended in 0.5 mL of RPMI 1640 medium containing 5% foetal bovine serum and 0.2% sodium azide at room temperature. The remaining material was washed twice in the same medium, re-suspended in RNAlater® solution and stored at -20°C for total RNA isolation.

Total RNA extraction, at least 0.5 mL, of each cell suspension containing 2 × 10^6^ cell/mL of good viability was used for lymphoma cell lines.

### Quantitative real-time RT-PCR (qRT-PCR)

The total RNA was isolated from both cell pellets and RNAlater® suspensions, as recommended by Dunmire *et al.*[20], using the RNeasy Mini Kit according to the manufacturer’s instructions. To avoid genomic DNA contamination, on-column DNase digestion with the RNase-Free DNase set was performed. The total RNA concentration and quality were measured using a Nanodrop ND-1000 spectrophotometer (Nanodrop Technologies, Wilmington, DE) and by denaturing gel electrophoresis. First-strand cDNA was synthesised from 200 ng of total RNA using the High Capacity cDNA Reverse Transcription Kit and the QuantiTect Reverse Transcription Kit for FNAs and lymphoma cell lines, respectively, according to the manufacturer’s protocol. The generated cDNA was then used as the template for qRT-PCR in a LightCycler 480 Instrument (Roche Diagnostics) using standard PCR conditions. The qRT-PCR reactions consisted of 1× LightCycler 480® Probe Master, 300 or 600 nM forward and reverse primers (the primer combination and final concentrations were optimised during the assay setup), 100 nM human UPL probe and 2.5 μL of 25-fold-diluted cDNA. Primer pairs and human UPL probes for MMP-2, MMP-9, MT1-MMP, TIMP-1, TIMP-2, RECK and VEGF-A amplification were described previously [6,10]. In the present study, VEGF-164, the VEGF-A splice variant markedly expressed in dogs and highly conserved among species [21] was also considered. Canine VEGF-164 was amplified using the primer pair 5′-CGT GCC CAC TGA GGA GTT-3′ (forward) and 5′-AAG GCC CAC AGG GAT TTT CT-3′ (reverse) and human UPL probe #9. The concentration of each set of primers was optimised to efficiently amplify its target. Agarose gel electrophoresis confirmed the amplification of a single amplicon of the expected size. Calibration curves using a 4-fold serial dilution of a cDNA pool revealed PCR efficiencies comprised in the range of acceptance (90-110%) and error values < 0.2 (see Table 1). The canine transmembrane BAX inhibitor motif containing 4 (TMBIM4 or CGI-119) [22] was chosen as reference gene for the absence of statistically significant differences in its expression profile between the healthy and pathologic samples. Moreover its amplification efficiency was approximately equal to those of the target genes. The relative quantification of messenger RNA was performed using the ∆∆Ct method [23]. The relative quantification values were ultimately expressed as arbitrary units.

**Table 1:** qRT-PCR assay parameters: primer concentration, efficiency, linearity and dynamic range

**Genes**	**Primer concentration (nM)**	**Efficiency (%)**	**Error**	**Dynamic range (Cp)**
TMBIM4	F600/R300	90.2	0.00291	23.90 – 32.39
MMP-2	F600/R600	100.2	0.03450	25.57 – 32.68
MMP-9	F300/R300	99.8	0.05740	30.50 – 36.28
MT1-MMP	F600/R600	100.1	0.02890	24.64 – 31.68
TIMP-1	F600/R300	100.0	0.03110	25.24 – 31.25
TIMP-2	F300/R300	103.4	0.02890	24.50 – 31.38
RECK	F600/R300	101.0	0.06310	27.05 – 33.78
VEGF-A	F300/R300	102.8	0.01020	26.56 – 32.49
VEGF-164	F300/R300	99.8	0.02690	28.54 – 35.19

F, forward primer; R, reverse primer.

### Immunocytochemical analysis

The protein expression levels of MMP-2, MMP-9 and VEGF-A of the lymphoma samples and the cell lines were evaluated by immunocytochemistry. The cellular suspension designated for immunocytochemical analysis was prepared by cytospin and fixed with acetone and methanol. Sections of five normal lymph nodes were used for immunohistochemical analysis. The primary antibody incubation step was performed by an automated system for all antibodies (Ventana Medical Systems). The antibodies used in this study were the following: anti-human MMP-9, Clone C-TERM (1:200; Millipore S.p.A, Milan, Italy); anti-human MMP-2, Clone Ab-7 (1:100; Thermo Fisher Scientific Inc., Kalamazoo, Michigan, USA); and anti-human VEGF-A, Clone A-20 – sc:152 (1:200; Santa Cruz Biotechnology, Inc., Santa Cruz, California, USA). The Ventana ES automated immunohistochemistry system was used for the remainder of the staining procedure, including the incubation with a biotinylated anti-mouse secondary antibody, the diaminobenzidine substrate and a haematoxylin counterstain. Positive controls for MMP-2 and MMP-9 consisted of fibroblasts and granulocytes in sections of canine mammary tumours [6]. The endothelial cells in normal skin were used as positive control for VEGF-A [11]. Negative control slides were incubated with isotype-matched immunoglobulin in parallel with each staining batch to confirm the specificity of the antibodies. Immunoreactivity for MMP-2, MMP-9 and VEGF-A was scored in the following manner: 0: <10% positive cells, 1: 10–24% positive cells; 2: 25–49% positive cells; 3: >50% positive cells. The image analysis system included an Olympus BX51 microscope and a software analysis (analySIS, Soft imaging system, Münster, Germany).

### Gelatin zymography

MMP-2 and MMP-9 activity of the lymphoma samples was studied by zymography. Briefly, the cells were lysate and centrifuged at 1500 rpm for 10 min, and the protein concentration of the supernatant was measured. The sample protein concentration was adjusted to 1 mg/ml, and 5 μl was diluted 1:1 in sample buffer; the final 10 μl sample was subjected to electrophoresis on an 8% SDS-PAGE gel copolymerized with 0.1% gelatin. Following electrophoresis, the gel was incubated for 1 h at room temperature in a 2.5% Triton X-100 solution and then at 37°C for 16 h in 0.5 M Tris–HCl buffer, pH 7.4, with 10 mM CaCl2. The gels were stained with 0.1% Coomassie Brilliant Blue R-250 and de-stained with 30% methanol and 10% acetic acid. Gelatinolytic activities were detected as unstained bands against the background of Coomassie-stained gelatin. Culture medium conditioned by A2058 melanoma cells was used as a control to identify the pro-MMP-9 gelatinolytic band, whereas conditioned media from HT1080 fibrosarcoma cells was used for the active forms of MMP-2 and MMP-9 and small amounts of the pro-MMP-2 [24].

### Statistical analysis

The statistical analysis of the gene expression results was performed using Mann–Whitney test and Kruskal-Wallis test followed by Dunn’s post-test. A non-parametric Spearman correlation analysis was used to determine the potential relationship between target genes and between gene expression levels and percentage of neoplastic cells in B and T-cell lymphomas. GraphPad Prism 5 software (San Diego, California, USA) was used for all statistical evaluations. Statistical significance was set at *p*<0.05. Finally, Grubbs’ test was used to identify potential outliers.

## Results

### Clinical results

Forty-seven dogs with multicentric lymphoma were enrolled. Based on cytological and flow cytometric evaluation, there were 26 B-cell lymphomas (22 high-grade, HG, and 4 low-grade, LG), and 21 T-cell lymphomas (13 HG and 8 LG).

### qRT-PCR

The gene expression results for healthy control lymph nodes, B-cell and T-cell lymphomas, and cells lines are summarised in Table 2. MMP-2 and TIMP-2 mRNA levels in the healthy control lymph nodes were significantly higher than in lymphomas (*p*<0.05). Significantly higher MMP-9 and TIMP-1 mRNA expression levels were observed in T-cell lymphomas compared to B-cell lymphomas and healthy controls (*p*<0.05). Furthermore, a statistically significant higher MT1-MMP mRNA amount was observed in T-cell lymphomas compared to B-cell lymphomas (*p*<0.05). MT1-MMP mRNA levels were also significantly higher in control lymph nodes than in B-cell lymphomas (*p*<0.05). When considering the tissue inhibitor RECK, statistically significant differences were observed between B-cell and T-cell lymphomas, and between T-cell lymphomas and healthy controls (*p*<0.05).

**Table 2:** MMP-9, MMP-2, MT1-MMP, TIMP-1, TIMP-2, VEGF-A, VEGF-164 and RECK mRNA expression in control lymph nodes, B-cell and T-cell lymphomas, CLBL-1 and OSW lymphoma cell lines

**Genes**	**Control lymph nodes**	**B-cell lymphoma**	**T-cell lymphoma**	**CLBL-1**	**OSW**
MMP-2	0.96 ± 0.23^a,b^	0.24 ± 0.28	0.16 ± 0.29	0.00	0.00
TIMP-2	0.85 ± 0.34^a,b^	0.15 ± 0.20	0.16 ± 0.29	0.00	0.82
MT1-MMP	0.61 ± 0.17^a^	0.11 ± 0.11^c^	0.94 ± 0.99	0.04	4.11
MMP-9	0.13 ± 0.11^b^	0.12 ± 0.04 ^c^	0.69 ± 0.11	0.76	1128.35
TIMP-1	0.15 ± 0.09^b^	0.16 ± 0.22 ^c^	0.66 ± 0.75	0.72	2.38
RECK	0.29 ± 0.12^b^	0.06 ± 0.12^c^	0.87 ± 1.12	4.32	19.56
VEGF-A	0.37 ± 0.19^b^	0.71 ± 0.98	0.89 ± 1.37	4.87	1.42
VEGF-164	0.57 ± 0.23	0.63 ± 0.92	0.90 ± 1.38	7.99	3.18

Data are expressed as relative quantification values (arbitrary units, mean ± SD).

^a, b, c^ Significant differences between control lymph nodes and B-cell lymphoma, control lymph nodes and T-cell lymphoma, B-cell lymphoma and T-cell lymphoma, respectively (Kruskal-Wallis test followed by Dunn’s post test; *p* < 0.05).

B-cell and T-cell lymphomas had similar VEGF-A and VEGF-164 expression profiles, moreover T-cell lymphomas showed significantly higher expression of VEGF-A with respect to controls (*p*<0.05).

Interestingly, higher MMP-9, MT1-MMP, VEGF-A, VEGF-164 and TIMP-1 mRNA expressions were observed in HG T-cell lymphomas compared to LG T-cell lymphomas, although the differences were not statistically significant (Table 3). The same analysis was not performed in B-cell lymphomas because of the low number of LG cases included in the study.

**Table 3:** MMP-9, MMP-2, MT1-MMP, TIMP-1, TIMP-2, VEGF-A, VEGF-164 and RECK mRNA expression in high grade and low grade T-cell lymphomas and HG B-cell lymphomas

**Genes**	**T-cell lymphoma**		**B-cell lymphoma**
**tt**	**High grade**	**Low grade**	**High grade**
MMP-2	0.18 ± 0.22	0.17 ± 0.20	0.26 ± 0.29
MMP-9	0.82 ± 0.15 ^a^	0.12 ± 0.28	0.01 ± 0.03
MT1-MMP	1.01 ± 1.08 ^a^	0.81 ± 0.86	0.10 ± 0.11
TIMP-1	1.07 ± 1.98 ^a^	0.63 ± 0.92	0.13 ± 0.21
TIMP-2	0.15 ± 0.28	0.16 ± 0.32	0.14 ± 0.19
RECK	0.93 ± 1.26 ^a^	1.67 ± 2.76	0.06 ± 0.12
VEGF-A	1.21 ± 1.65	0.35 ± 0.43	0.67 ± 0.99
VEGF-164	1.11 ± 0.92	0.39 ± 0.88	0.49 ± 0.65

Data are expressed as relative quantification values (arbitrary units, mean ± SD)

^a^Significant difference between high grade T-cell and B-cell lymphomas (Mann–Whitney test; *p* < 0.05).

The expressions of MMP-9, MT1-MMP, TIMP-1 and RECK were significantly higher in HG T-cell lymphomas compared to HG B-cell lymphomas (*p*<0.05) (Table 3). Although not statistically significant, VEGF-A and VEGF-164 mRNA expressions showed the same trend. Significant correlations between MMP-2 and TIMP-2 (*p*<0.001; Spearman r=0.82) and between MMP-9 and VEGF-A (*p*<0.01; Spearman r=0.67) were found in T-cell lymphomas. No statistically significant correlations were found between gene expression and the percentage of neoplastic cells in B-cell and T-cell lymphomas.

The gene expression results for cell lines are summarised in Table 2. A higher MMP-9 mRNA expression was observed in OSW compared to CLBL-1; whereas MMP-2 mRNA was undetectable in both cell lines. MT1-MMP and TIMP-1 mRNAs were detected in OSW, but no results were obtained for CLBL-1. VEGF-A and VEGF-164 transcripts were detected in both cell lines. In CLBL-1 and OSW, TIMP-2 and TIMP-1 mRNA expressions were close to 0, whereas RECK was detected.

### Immunocytochemical analysis

**Figure 1:**
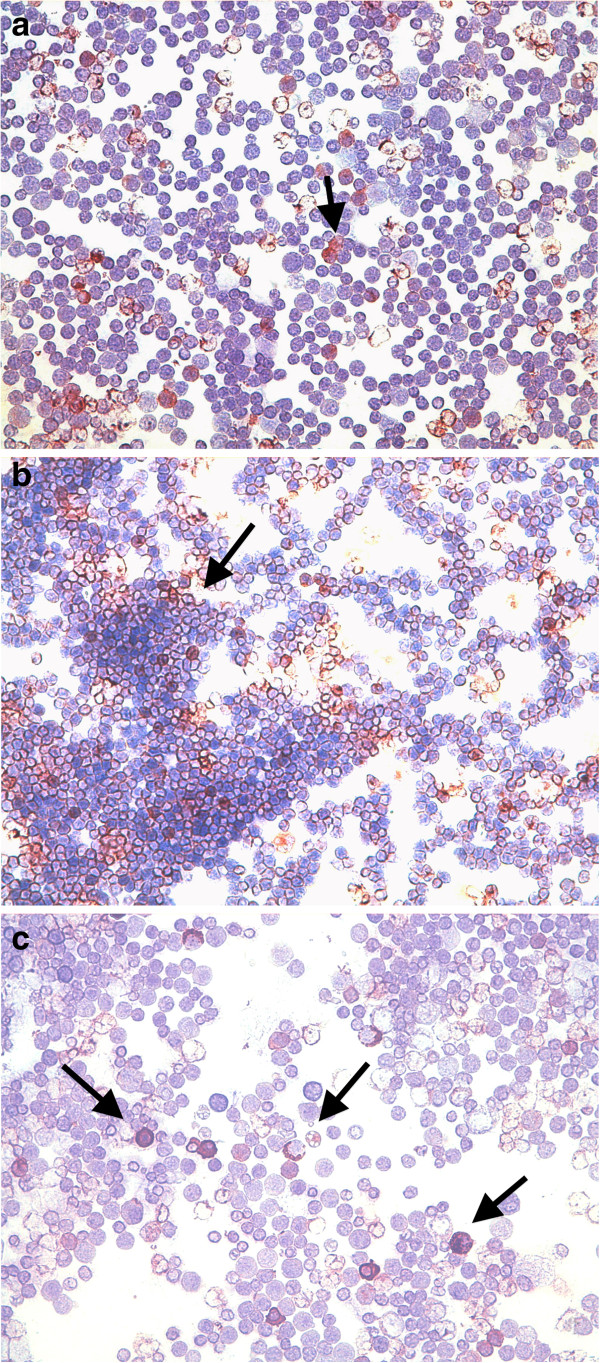
**B-cell lymphoma FNAs.** (**a**) low number of positive immunoassayed lymphoid tumour cells for MMP-2 antibody (arrow); (**b**) numerous positive immunoassayed lymphoid tumour cells for MMP-9 antibody (arrow); (**c**) plasma cells and low percentage of immunoassayed lymphoid tumour cells for VEGF-A antibody (arrows). Immunocytochemistry, 400×.

**Figure 2:**
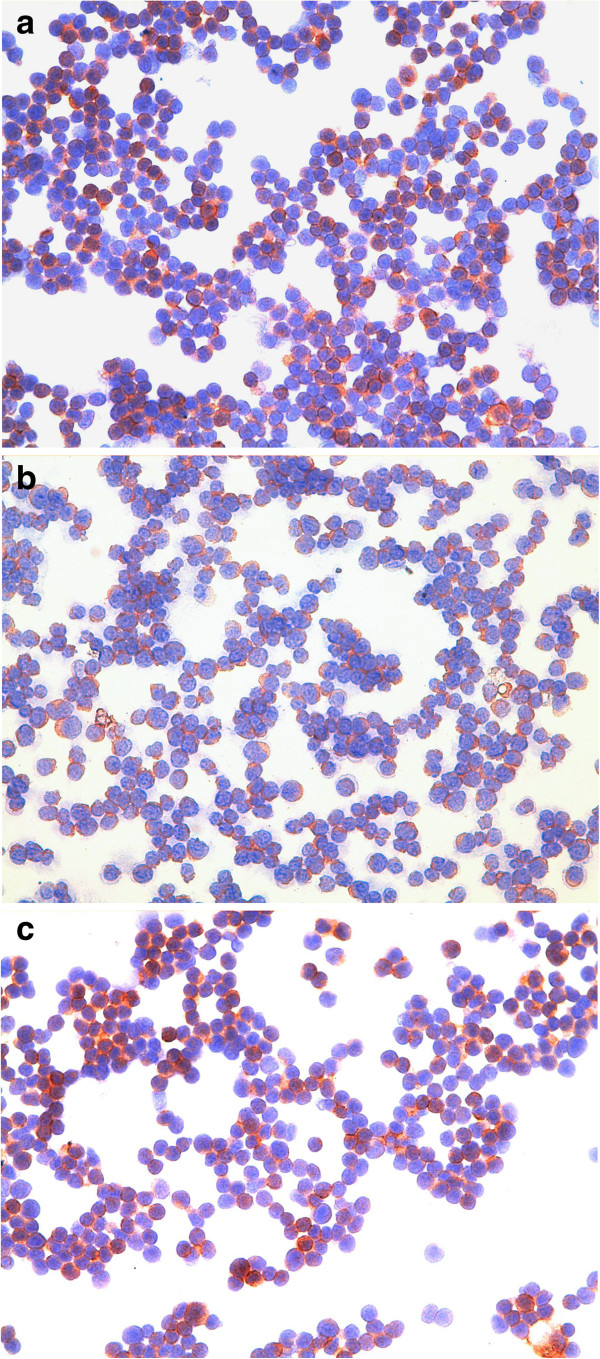
**T-cell lymphoma FNAs.** Intense and widespread positive immunoassayed lymphoid tumour cells for (**a**) MMP-2 antibody, (**b**) MMP-9 antibody and (**c**) VEGF-A antibody. Immunocytochemistry, 400×.

**Figure 3:**
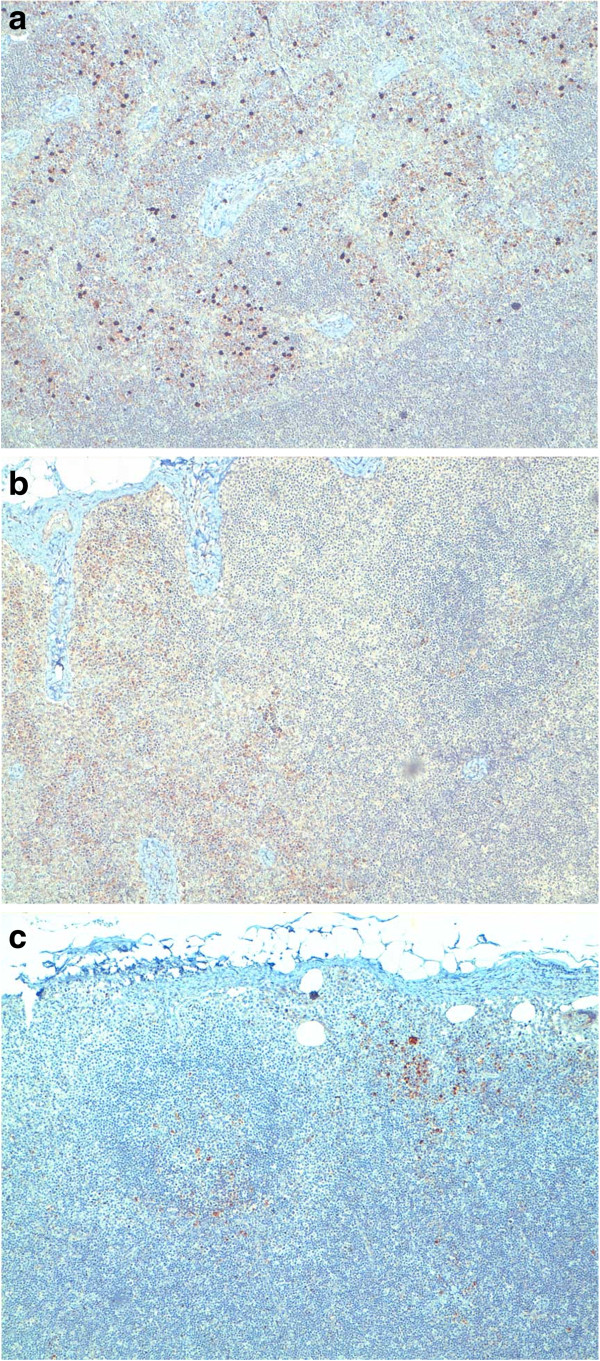
**Control lymph nodes.** Scattered positive immunoassayed lymphocytes for MMP-2 (**a**), MMP-9 (**b**) and VEGF-A (**c**). Immunohistochemistry, 200×.

**Figure 4:**
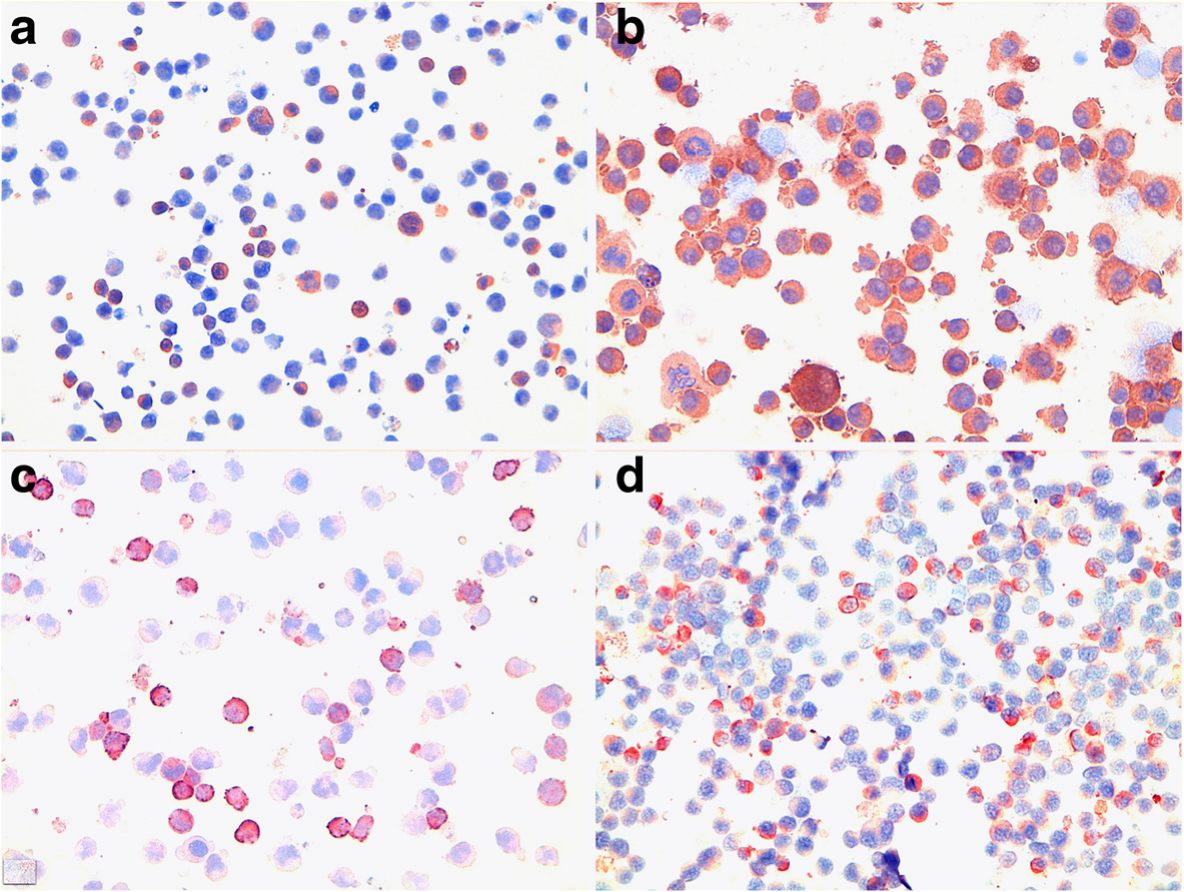
**Cell lines. a**) low number of positive immunoassayed cells in CLBL-1 for MMP-9 antibody **b**) intense and widespread positive immunoassayed cells in OSW for MMP-9 antibody, **c**) low number of positive immunoassayed cells in CLBL-1 for VEGF-A antibody, **d**) low number of positive immunoassayed cells in OSW for VEGF-A antibody. Immunocytochemistry, 400×.

The immunocytochemical expression of MMP-9 was grade 3 in 16 T-cell lymphomas (13 HG and 3 LG) and grade 2 in the remaining T-cell lymphomas (5 LG), whereas in 21 B-cell lymphomas the expression was grade 1 (18 HG and 3 LG) and grade 2 in the remaining cases (4 HG and 1 LG). MMP-2 expression was grade 2 in all T-cell lymphomas; grade 1 in 19 HG B-cell lymphomas and grade 0 in the remaining 7 B-cell lymphomas (3 HG and 4 LG). VEGF-A labelling was scored grade 2 in 19 T-cell lymphomas (13 HG and 6 LG), grade 1 in 2 LG T-cell lymphomas and all the B-cell lymphomas. Representative images of MMP-9, MMP-2 and VEGF-A immunolabelling in lymphomas are shown in Figures 1 and 2. The expression of MMP-2, MMP-9 and VEGF-A was grade 0 in the 5 control lymph nodes (Figure 3a, 3b and 3c). MMP-9 was highly expressed in OSW (92%) whereas expression was weaker in CLBL-1 (19%) (Figure 4a and 4b). No MMP-2 expression was found in both cell lines. CLBL-1 and OSW showed VEGF-A positive immunolabelling (22%) (Figure 4c and 4d). The mean immunocytochemical scores are shown for B-cell lymphomas, T-cell lymphomas and control lymph nodes in Table 4.

**Table 4:** Immunocytochemical scores for MMP-9, MMP-2 and VEGF-A in B-cell lymphomas, T-cell lymphomas and control lymph nodes expressed as mean (min-max)

	**MMP-9**	**MMP-2**	**VEGF-A**
**HG B-cell lymphomas**	22.1% (13–35)	16.9% (3–22)	17.3% (11–23)
**LG B-cell lymphomas**	15.7% (11–27)	4.2% (2–7)	13.8% (12–17)
**HG T-cell lymphomas**	79.8% (56–89)	38.4% (29–45)	44.7% (38–48)
**LG T-cell lymphomas**	41.3% (25–63)	30.2 (26–33)	30.2% (18–37)
**Control lymph nodes**	4.2% (1–7)	0.8% (0–2)	0.8% (0–2)

### Gelatine zymography

Both latent and active forms of MMP-2 and MMP-9 were undetectable at gelatine zymography in lymphoma samples.

## Discussion

In human haematologic malignancies, VEGF and different enzymes involved in ECM remodelling, including MMPs, are considered molecules for early diagnosis and prognostic assessment [25-27]. Recently, we suggested a potential role of MMP-9, MT1-MMP, TIMP-1, TIMP-2 and VEGF in the pathogenesis of canine leukaemia [12]. In this study we investigated the role of different MMPs, their regulators and VEGF in canine lymphoma by assessing mRNA and protein expression profiles.

Higher MMP-9 protein and mRNA expression levels were observed in T-cell lymphomas compared to B-cell lymphomas and healthy control lymph nodes, indicating that MMP-9 may be associated with tumour phenotype. In human non-Hodgkin lymphoma a trend toward an unfavourable prognosis and MMP-9 expression has been demonstrated [28]. When we compared HG and LG lymphomas, MMP-9 overexpression was found in HG T-cell lymphomas respect with LG T-cell lymphomas. HG T-cell lymphomas are characterized by organ invasion and bone marrow infiltration, and are considered highly aggressive [2]. Biologically, T-cells are able to migrate across ECM barriers during the inflammatory process towards target tissues and the activation of MMP-9 causes alteration of adjacent connective tissues and degradation of collagen type IV [29]. The immunohistochemical results in normal lymph nodes sections identified a low number of MMP-9 positive cells in area compatible with germinal centres and paracortex, indicating that normal B-cells are also able to express a minimum amount of MMP-9. However, T-cells might be able to increase MMP-9 production during the neoplastic transformation more than B-cells. MMP-9 results in CLBL-1 and OSW cells additionally confirmed the different MMP-9 expression both at the mRNA and protein level in B- and T-cell lymphomas. The equivalent expression value of the T-cell lymphoma samples and the CLBL-1 cell line could be due to a difference in primary material and a cell line. Nevertheless, the very high expression in the OSW cells strongly supports the hypothesis for T-cell expression. This data also seems to exclude a possible interference of the microenvironment in MMP-9 regulation.

Significantly higher levels of TIMP-1 mRNA were observed in T-cell lymphomas compared to B-cell lymphomas and controls. This same result was obtained also *in vitro*, as the T-cell lymphoma cell line (OSW) showed about 3-fold TIMP-1 mRNA higher amount than B-cell lymphoma cell line (CLBL-1). Overall, this experimental evidence supports the hypothesis that MMP-9 and TIMP-1 may act in concert in canine T-cell lymphoma. MMP-9 is frequently expressed and secreted with TIMP-1 by canine neoplastic cells [6,12]. These molecules are associated with a more aggressive clinical behaviour in human lymphoma, and they appear to exert their influence through two different mechanisms: MMP-9 causes ECM degradation, whereas TIMP-1 shows an anti-apoptotic action [30,31]. The same may hold true in dog where HG T-cell lymphomas showed a higher TIMP-1 mRNA expression than LG.

MMP-2 was negatively immunolabelled in lymphocytes of control lymph nodes, whereas B-cell and T-cell lymphomas exhibited minimal to moderate amount of MMP-2. Interestingly, mRNA expression was significantly higher in control dogs with respect to dogs with lymphoma. The mRNA expression in control lymph nodes may be altered by the presence of a variable number of cells that are normally found within the node and in the perinodal tissue, such as fibroblasts and plasmacells, constitutively expressing MMP-2 [6]. Furthermore, in different solid tumours, variable MMP-2 protein amounts with insignificant expression of MMP-2 mRNA have been shown and correlated to feedback mechanisms, being able to shut off messenger expression after the secretion and/or binding of the protein [32]. A different post-transcriptional regulation of MMP-2 or indirect mechanisms of activation may be hypothesised in lymphomas. Hypoxia, macrophages, granulocytes and endothelial cells are known to activate MMP-2 in tumour invasion and angiogenesis but the specific mechanism of MMP-2 activation *in vivo* is yet not fully understood. Activation of proMMP-2 and concomitant induction of MT1-MMP mRNA expression has been found in human fibroblasts, endothelial cells, and breast carcinoma cells [33]. In T-cell lymphomas MT1-MMP mRNA levels were higher compared to B-cell lymphomas and in HG T-cell lymphomas with respect to LG lymphomas. This phenomenon was also seen in the two cell lines. This result identifies a different biological behaviour for this transcript based on the phenotype and morphological features. Besides MT1-MMP, other auxiliary components such as TIMP-2 and integrins are required for activation of pro-MMP-2. It has been shown that TIMP-2 plays a critical role in MMP-2 activation on the cell surface by binding to MT1-MMP [5]. Interestingly, in T-cell lymphomas MMP-2 qRT-PCR analysis revealed a significant positive correlation with TIMP-2. Unfortunately we were not able to perform immunohistochemistry for MT1-MMP and TIMP-2 in the present work.

We also performed gelatine zymography in T-cell and B-cell lymphomas to investigate the activity of MMP-2 and MMP-9, but no catalytic activity was detected. While it is relatively easy to detect MMPs in media obtained from cell culture, the extraction and analysis of MMPs and TIMPs from cells are much more difficult and the number of cells might influence detection [34]. A possible limit in the study is the lack of lymphoma tissue; so far, further experiments should be directed to identify the catalytic activity of MMP-9 and MMP-2 in B-cell and T-cell lymphomas.

In both T- and B-cell lymphomas, we observed VEGF-A expression at the mRNA and protein level. HG T-cell lymphomas showed higher VEGF-A mRNA expression compared with LG T-cell lymphomas and moreover the mRNA VEGF-A results were correlated with MMP-9 results in T-cell lymphomas. These data appear to be in accordance with our previous work, in which we reported a close relationship between MMP-9 and VEGF plasmatic levels in canine lymphomas [13]. We also observed the same results in canine mast cell tumours, in which the release of VEGF by mast cells is correlated with higher MMP-9 production [10]. Indeed, the feedback activation between MMP-9 and VEGF is implicated in the angiogenic switch. In fact VEGF-A is known to be the most important mediator of angiogenesis. In the present study we could not associate VEGF-A protein and gene expression results to an increased microvessel density but this was demonstrated to be not significant in a precedent work [35]. Interestingly, cell lines showed a similar result for VEGF-A and VEGF-164 which was fairly high in both cell lines compared to the primary material. Furthermore, the different results for gene expression and protein data in normal lymph nodes may confirm an overexpression of VEGF-A in canine lymphoma.

## Conclusion

In conclusion, our data provide new information on the expression of different MMPs and VEGF in canine lymphoma. Further efforts should be directed towards clarifying the detailed molecular mechanisms of MMPs, such as signal transduction and proteolytic activity. In human non-Hodgkin lymphoma, functional VEGF polymorphisms, which have an effect on the regulation of gene expression, contribute to the differences between individuals. Future investigations will be directed in this direction in canine lymphoma. The results from this study also indicate that differences between lymphoma subtypes must be taken into account in the selection of the most suitable canine patients for trials with anti-angiogenic agents and MMPs inhibitors.

## Abbreviations

ECM: Extracellular matrix; MMP: Matrix metalloproteinases; MT1-MMP: Membrane type 1 matrix metalloproteinase; TIMP: Tissue inhibitors of matrix metalloproteinase; VEGF: Vascular endothelial growth factor; qRT-PCR: Quantitative real-time RT-PCR; UPL: Universal Probe Library; TMBIM4 or CGI-119: Transmembrane BAX inhibitor motif containing 4; RECK: Reversion-inducing cysteine-rich-protein with Kazal-motifs; FNA: Fine-needle aspirate.

## Competing interests

The authors declare that they have no competing interests.

## Authors’ contributions

AA performed the gene expression and the immunocytochemistry experiments, the statistical analysis and drafted the manuscript. MG designed and performed the gene expression experiments, performed the statistical analysis and helped to draft the manuscript. MEG, FR, SC, BCR and SHE provided the tumour and control samples, participated in the design and coordination of the study and helped to draft the manuscript. MC provided the tumour and control samples. MD participated in the design and coordination of the study and helped to draft the manuscript. LA conceived of the study, participated in its design and coordination. All authors read and approved the final manuscript.
